# *N*-(2-Chloro­acet­yl)glycine

**DOI:** 10.1107/S1600536813028997

**Published:** 2013-10-26

**Authors:** Yu-Cheng Zhang, Xiu-Qin Zhang, Kai Wang, Qiang Chen

**Affiliations:** a School of Materials Science and Engineering, Changzhou University & High Technology, Research Institute of Nanjing University, Changzhou 213162, Jiangsu, People’s Republic of China; b High Technology Research Institute of Nanjing University, Changzhou 213162, Jiangsu, People’s Republic of China

## Abstract

The title compound, C_4_H_6_ClNO_3_, crystallizes with two independent mol­ecules (*A* and *B*) in the asymmetric unit. In each mol­ecule, there are N—H⋯O and N—H⋯Cl hydrogen bonds. Both mol­ecules are relatively planar, with the mean plane of the acetamide [N—C(=O)C] group being inclined to the mean plane of the acetate group [C—C(=O)O] by 9.23 (13)° in mol­ecule *A* and 6.23 (12)° in mol­ecule *B*. In the crystal, adjacent mol­ecules are linked by O—H⋯O hydrogen bonds and weak C—H⋯O contacts forming –*A*–*A*–*A*– and –*B*–*B*–*B*– parallel chains propagating along the *a*-axis direction.

## Related literature

For the use of the title compound as an inter­mediate in the synthesis of polydespipeptides and their copolymers, which have a wide range of biomedical properties, see: Feng *et al.* (2010 ▶). For the synthetic procedure, see: Allmendenger *et al.* (1988 ▶). For bond-length data, see: Allen *et al.* (1987 ▶). For the crystal structure of (2,2,2-tri­chloro­acte­yl)glycine, see: Dou *et al.* (1995 ▶).
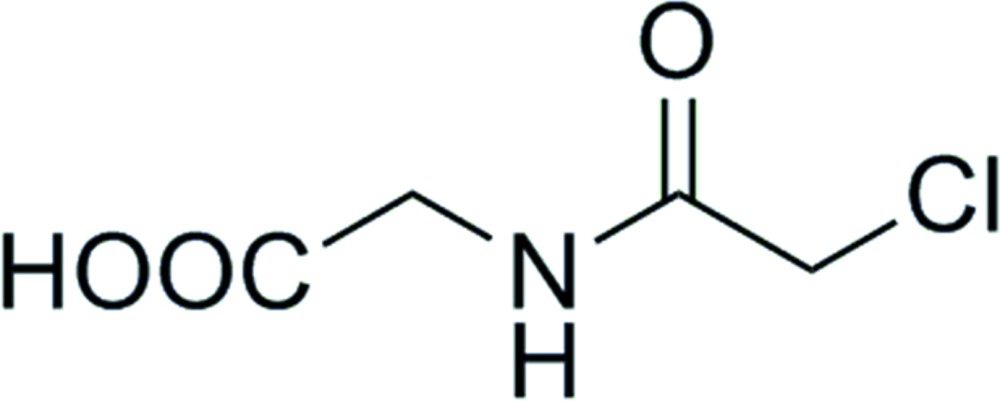


## Experimental

### 

#### Crystal data

C_4_H_6_ClNO_3_
*M* *_r_* = 151.55
Monoclinic, 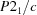

*a* = 18.001 (4) Å
*b* = 7.6371 (17) Å
*c* = 9.372 (2) Å
β = 105.025 (3)°
*V* = 1244.4 (5) Å^3^
*Z* = 8
Mo *K*α radiation
μ = 0.54 mm^−1^
*T* = 296 K
0.28 × 0.22 × 0.15 mm

#### Data collection

Enraf–Nonius CAD-4 diffractometer
Absorption correction: ψ scan (North *et al.*, 1968 ▶) *T*_min_ = 0.863, *T*_max_ = 0.923
9419 measured reflections
2419 independent reflections
2143 reflections with *I* > 2σ(*I*)
*R*_int_ = 0.044
3 standard reflections every 120 reflections intensity decay: 1%

#### Refinement

*R*[*F* ^2^ > 2σ(*F* ^2^)] = 0.038
*wR*(*F* ^2^) = 0.103
*S* = 1.07
2419 reflections
167 parameters
2 restraints
H-atom parameters constrained
Δρ_max_ = 0.29 e Å^−3^
Δρ_min_ = −0.30 e Å^−3^

### 

Data collection: *CAD-4 Software* (Enraf–Nonius, 1985 ▶); cell refinement: *CAD-4 Software*; data reduction: *XCAD4* (Harms & Wocadlo, 1995 ▶); program(s) used to solve structure: *SHELXS97* (Sheldrick, 2008 ▶); program(s) used to refine structure: *SHELXL97* (Sheldrick, 2008 ▶); molecular graphics: *SHELXTL* (Sheldrick, 2008 ▶); software used to prepare material for publication: *SHELXTL*.

## Supplementary Material

Crystal structure: contains datablock(s) I, zhang. DOI: 10.1107/S1600536813028997/su2655sup1.cif

Structure factors: contains datablock(s) I. DOI: 10.1107/S1600536813028997/su2655Isup2.hkl

Supplementary material file. DOI: 10.1107/S1600536813028997/su2655Isup3.cml

Additional supplementary materials:  crystallographic information; 3D view; checkCIF report

## Figures and Tables

**Table 1:** Hydrogen-bond geometry (Å, °)

*D*—H⋯*A*	*D*—H	H⋯*A*	*D*⋯*A*	*D*—H⋯*A*
N1—H1′⋯Cl1	0.86	2.44	2.9422 (18)	118
N1—H1′⋯O2	0.86	2.24	2.618 (2)	106
N2—H2′⋯Cl2	0.86	2.46	2.9450 (18)	116
N2—H2′⋯O4	0.86	2.22	2.619 (2)	108
O1—H1⋯O3^i^	0.82	1.84	2.647 (2)	166
O5—H5⋯O6^ii^	0.82	1.85	2.657 (2)	167
C4—H4*B*⋯O2^ii^	0.97	2.57	3.080 (3)	113
C8—H8*B*⋯O4^i^	0.97	2.57	3.099 (3)	114

Symmetry codes: (i) 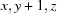; (ii) 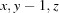.

**Fig. 1.**
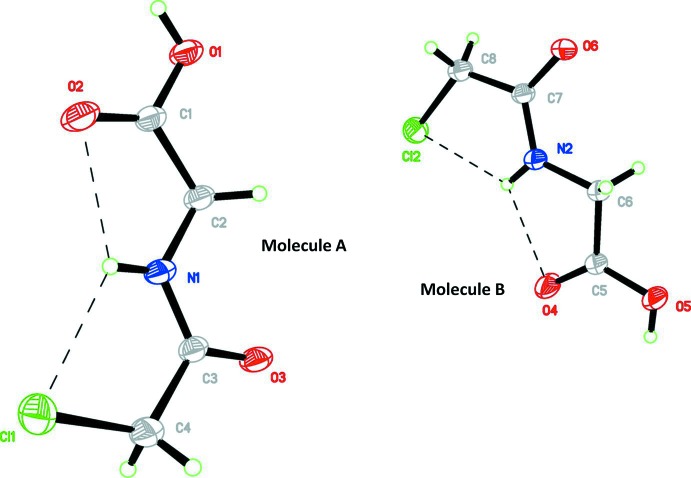
The molecular structure of the two independent molecules (A and B) of the title compound, with atom labelling. Displacement ellipsoids are drawn at the 50% probability level. The intramolecular N-H···O and N-H···Cl hydrogen bonds are shown as dashed lines (see Table 1 for details).

**Fig. 2.**
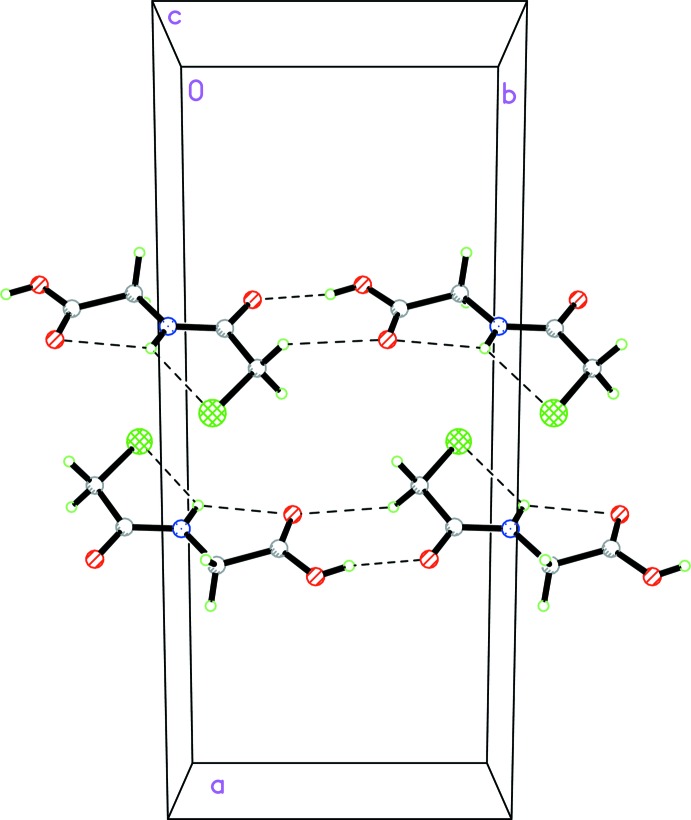
A view along the c axis of the crystal packing of the title compound. The various hydrogen bonds and short contacts are shown as dashed lines (see Table 1 for details; H atoms not involved in hydrogen bonding have been omitted for clarity).

## supplementary crystallographic information

### 1. Comment

The title compound is an important organic intermediate which has been used to synthesis polydespipeptides and their copolymers, that have a wide range of biomedical applications such as, tissue engineering, drug delivery, and the synthesis of artificial skin (Feng *et al.*, 2010). Herein we report on its crystal structure.

The two indepedent molecules of the title compound are shown in Fig. 1. In each molecule the NH hydrogen atom is hydrogen bonded to the adjacent O and Cl atoms (Table 1 and Fig. 1). Both molecules are planar with a maximum deviation of and , respectively for the mean planes of the non-H atoms.

The bond lengths (Allen *et al.*, 1987) and angles are within normal ranges. The bond distances are similar to those observed for the trichloro derivative, (2,2,2-Trichloroacteyl)glycine (Dou *et al.*, 1995), which also crystallizes with two independent molecules in the asymmetric unit.

In each molecule of the title compound the NH hydrogen atom is hydrogen bonded to the adjacent O and Cl atoms (Table 1 and Fig. 1). Both molecules are relatively planar, with the mean plane of the acetamide [N-C(═O)C] group being inclined to the mean plane of the acetate group [C-C(═O)O] by 9.23 (13) ° in molecule A and 6.23 (12) ° in molecule B.

In the crystal, adjacent similar molecules are linked by O—H···O and hydrogen bonds and weak C-H···O contacts forming -A-A-A- and -B-B-B- parallel chains propagating along the a axis direction (Table 1 and Fig. 2).

### 2. Experimental

The title compound was prepared by a method reported in the literature (Allmendenger *et al.*, 1988). A solution of NaOH (93.75 ml, 4 mol/*L*) and 2-chloroacetyl chloride (22.6 ml, 0.3 mol) were added separately and slowly to a solution of *N*-(chloroacetyl)-glycine sodium salt [prepared by mixing NaOH (13.2 g, 0.3 mol) and glycine (25 g, 0.3 mol) at pH = 11 in an ice bath]. After stirring for 2 h at room temperature, HCl was added to adjust the pH to 2. Then ethyl acetate was added, and the solvent filtered. The organic phase was evaporated on a rotary evaporator and the title compound was obtained. Colourless block-like crystals were obtained by slow evaporation of an ethyl acetate solution for 5 days at room temperature.

### 3. Refinement

All H atoms were positioned geometrically and constrained to ride on their parent atoms: N—H = 0.86 Å, O—H = 0.82 Å, C-H = 0.97 Å with *U*_iso_(H) = 1.5*U*_eq_(O) and = 1.2*U*_eq_(C), while for the NH H atoms *U*_iso_(H) was refined.

### Crystal data

**Table:**

C_4_H_6_ClNO_3_	*F*(000) = 624
*M**_r_* = 151.55	*D*_x_ = 1.618 Mg m^−^^3^
Monoclinic, *P*2_1_/*c*	Mo *K*α radiation, λ = 0.71073 Å
Hall symbol: -P 2ybc	Cell parameters from 4385 reflections
*a* = 18.001 (4) Å	θ = 3.5–27.1°
*b* = 7.6371 (17) Å	µ = 0.54 mm^−^^1^
*c* = 9.372 (2) Å	*T* = 296 K
β = 105.025 (3)°	Block, colourless
*V* = 1244.4 (5) Å^3^	0.28 × 0.22 × 0.15 mm
*Z* = 8	

### Data collection

**Table:**

Enraf–Nonius CAD-4 diffractometer	2143 reflections with *I* > 2σ(*I*)
Radiation source: fine-focus sealed tube	*R*_int_ = 0.044
Graphite monochromator	θ_max_ = 26.0°, θ_min_ = 2.3°
phi and ω scans	*h* = −22→22
Absorption correction: ψ scan (North *et al.*, 1968)	*k* = −7→9
*T*_min_ = 0.863, *T*_max_ = 0.923	*l* = −10→11
9419 measured reflections	3 standard reflections every 120 reflections
2419 independent reflections	intensity decay: 1%

### Refinement

**Table:**

Refinement on *F*^2^	Primary atom site location: structure-invariant direct methods
Least-squares matrix: full	Secondary atom site location: difference Fourier map
*R*[*F*^2^ > 2σ(*F*^2^)] = 0.038	Hydrogen site location: inferred from neighbouring sites
*wR*(*F*^2^) = 0.103	H-atom parameters constrained
*S* = 1.07	*w* = 1/[σ^2^(*F*_o_^2^) + (0.0508*P*)^2^ + 0.3706*P*] where *P* = (*F*_o_^2^ + 2*F*_c_^2^)/3
2419 reflections	(Δ/σ)_max_ = 0.001
167 parameters	Δρ_max_ = 0.29 e Å^−^^3^
2 restraints	Δρ_min_ = −0.30 e Å^−^^3^

### Special details

**Table:**

Geometry. All e.s.d.'s (except the e.s.d. in the dihedral angle between two l.s. planes) are estimated using the full covariance matrix. The cell e.s.d.'s are taken into account individually in the estimation of e.s.d.'s in distances, angles and torsion angles; correlations between e.s.d.'s in cell parameters are only used when they are defined by crystal symmetry. An approximate (isotropic) treatment of cell e.s.d.'s is used for estimating e.s.d.'s involving l.s. planes.
Refinement. Refinement of *F*^2^ against ALL reflections. The weighted *R*-factor *wR* and goodness of fit *S* are based on *F*^2^, conventional *R*-factors *R* are based on *F*, with *F* set to zero for negative *F*^2^. The threshold expression of *F*^2^ > σ(*F*^2^) is used only for calculating *R*-factors(gt) *etc*. and is not relevant to the choice of reflections for refinement. *R*-factors based on *F*^2^ are statistically about twice as large as those based on *F*, and *R*- factors based on ALL data will be even larger.

### Fractional atomic coordinates and isotropic or equivalent isotropic displacement parameters (Å^2^)

**Table:**

	*x*	*y*	*z*	*U*_iso_*/*U*_eq_	
C1	0.34036 (11)	0.8173 (2)	0.8783 (2)	0.0378 (4)	
C2	0.31910 (11)	0.6313 (2)	0.8351 (2)	0.0386 (4)	
H2A	0.2649	0.6123	0.8272	0.046*	
H2B	0.3288	0.6058	0.7401	0.046*	
C3	0.36784 (10)	0.3480 (2)	0.9359 (2)	0.0345 (4)	
C4	0.42198 (13)	0.2461 (3)	1.0570 (2)	0.0486 (5)	
H4A	0.4549	0.1752	1.0129	0.058*	
H4B	0.3918	0.1669	1.1003	0.058*	
C5	0.16319 (11)	0.1528 (2)	0.0418 (2)	0.0382 (4)	
C6	0.18026 (11)	0.3394 (2)	0.0134 (2)	0.0374 (4)	
H6A	0.2345	0.3637	0.0547	0.045*	
H6B	0.1678	0.3614	−0.0921	0.045*	
C7	0.13138 (10)	0.6218 (2)	0.06725 (18)	0.0338 (4)	
C8	0.07865 (12)	0.7218 (3)	0.1386 (2)	0.0463 (5)	
H8A	0.0449	0.7935	0.0636	0.056*	
H8B	0.1097	0.8005	0.2114	0.056*	
Cl1	0.48117 (3)	0.37273 (7)	1.20038 (5)	0.05350 (18)	
Cl2	0.02089 (3)	0.59470 (7)	0.22620 (6)	0.05211 (18)	
N1	0.36524 (9)	0.5190 (2)	0.94747 (17)	0.0395 (4)	
H1'	0.3955	0.5654	1.0245	0.057 (7)*	
N2	0.13446 (9)	0.4500 (2)	0.08162 (17)	0.0373 (4)	
H2'	0.1055	0.4000	0.1293	0.045 (6)*	
O1	0.30581 (9)	0.92850 (18)	0.77700 (15)	0.0498 (4)	
H1	0.3193	1.0283	0.8040	0.075*	
O2	0.38362 (11)	0.85625 (19)	0.99322 (17)	0.0647 (5)	
O3	0.32736 (8)	0.26765 (17)	0.82999 (15)	0.0456 (3)	
O4	0.12139 (12)	0.1121 (2)	0.1163 (2)	0.0708 (5)	
O5	0.19929 (9)	0.04173 (18)	−0.02249 (17)	0.0508 (4)	
H5	0.1878	−0.0585	−0.0052	0.076*	
O6	0.16923 (8)	0.70336 (17)	−0.00271 (15)	0.0430 (3)	

### Atomic displacement parameters (Å^2^)

**Table:**

	*U*^11^	*U*^22^	*U*^33^	*U*^12^	*U*^13^	*U*^23^
C1	0.0450 (10)	0.0252 (9)	0.0410 (9)	0.0029 (7)	0.0074 (8)	−0.0003 (7)
C2	0.0450 (10)	0.0246 (9)	0.0432 (10)	−0.0002 (7)	0.0058 (8)	−0.0014 (7)
C3	0.0409 (9)	0.0252 (9)	0.0403 (9)	−0.0002 (7)	0.0160 (8)	0.0000 (7)
C4	0.0645 (13)	0.0306 (10)	0.0441 (10)	0.0010 (9)	0.0024 (9)	−0.0013 (8)
C5	0.0492 (10)	0.0274 (9)	0.0415 (10)	−0.0003 (8)	0.0184 (8)	−0.0007 (7)
C6	0.0457 (10)	0.0268 (9)	0.0437 (9)	−0.0013 (7)	0.0186 (8)	−0.0002 (7)
C7	0.0403 (9)	0.0262 (9)	0.0335 (8)	−0.0012 (7)	0.0069 (7)	0.0001 (7)
C8	0.0573 (12)	0.0326 (10)	0.0559 (11)	−0.0008 (9)	0.0271 (10)	−0.0003 (9)
Cl1	0.0568 (3)	0.0499 (3)	0.0471 (3)	−0.0053 (2)	0.0013 (2)	−0.0023 (2)
Cl2	0.0526 (3)	0.0510 (3)	0.0600 (3)	−0.0071 (2)	0.0276 (2)	−0.0024 (2)
N1	0.0512 (9)	0.0226 (8)	0.0415 (8)	−0.0009 (7)	0.0063 (7)	−0.0007 (6)
N2	0.0466 (9)	0.0264 (8)	0.0435 (8)	−0.0025 (6)	0.0198 (7)	−0.0008 (6)
O1	0.0692 (10)	0.0246 (7)	0.0456 (7)	0.0040 (6)	−0.0030 (7)	0.0001 (5)
O2	0.0922 (12)	0.0266 (8)	0.0540 (9)	−0.0004 (7)	−0.0193 (9)	−0.0022 (6)
O3	0.0582 (8)	0.0242 (7)	0.0478 (7)	0.0002 (6)	0.0020 (6)	−0.0020 (5)
O4	0.1108 (14)	0.0295 (8)	0.1007 (13)	−0.0073 (8)	0.0786 (12)	−0.0046 (8)
O5	0.0690 (10)	0.0273 (7)	0.0676 (9)	0.0004 (6)	0.0386 (8)	−0.0027 (6)
O6	0.0562 (8)	0.0276 (7)	0.0514 (8)	0.0035 (6)	0.0250 (6)	0.0058 (6)

### Geometric parameters (Å, º)

**Table:**

C1—O2	1.192 (2)	C5—C6	1.496 (3)
C1—O1	1.305 (2)	C6—N2	1.441 (2)
C1—C2	1.499 (2)	C6—H6A	0.9700
C2—N1	1.443 (2)	C6—H6B	0.9700
C2—H2A	0.9700	C7—O6	1.230 (2)
C2—H2B	0.9700	C7—N2	1.318 (2)
C3—O3	1.232 (2)	C7—C8	1.504 (3)
C3—N1	1.312 (2)	C8—Cl2	1.772 (2)
C3—C4	1.507 (3)	C8—H8A	0.9700
C4—Cl1	1.770 (2)	C8—H8B	0.9700
C4—H4A	0.9700	N1—H1'	0.8597
C4—H4B	0.9700	N2—H2'	0.8598
C5—O4	1.192 (2)	O1—H1	0.8200
C5—O5	1.307 (2)	O5—H5	0.8200
			
O2—C1—O1	124.83 (17)	N2—C6—H6A	110.1
O2—C1—C2	122.81 (17)	C5—C6—H6A	110.1
O1—C1—C2	112.35 (15)	N2—C6—H6B	110.1
N1—C2—C1	107.95 (15)	C5—C6—H6B	110.1
N1—C2—H2A	110.1	H6A—C6—H6B	108.4
C1—C2—H2A	110.1	O6—C7—N2	122.95 (17)
N1—C2—H2B	110.1	O6—C7—C8	118.69 (16)
C1—C2—H2B	110.1	N2—C7—C8	118.35 (16)
H2A—C2—H2B	108.4	C7—C8—Cl2	116.19 (14)
O3—C3—N1	122.43 (17)	C7—C8—H8A	108.2
O3—C3—C4	118.78 (17)	Cl2—C8—H8A	108.2
N1—C3—C4	118.79 (16)	C7—C8—H8B	108.2
C3—C4—Cl1	115.73 (14)	Cl2—C8—H8B	108.2
C3—C4—H4A	108.3	H8A—C8—H8B	107.4
Cl1—C4—H4A	108.3	C3—N1—C2	123.89 (16)
C3—C4—H4B	108.3	C3—N1—H1'	116.8
Cl1—C4—H4B	108.3	C2—N1—H1'	119.1
H4A—C4—H4B	107.4	C7—N2—C6	123.51 (15)
O4—C5—O5	124.44 (18)	C7—N2—H2'	118.7
O4—C5—C6	122.84 (17)	C6—N2—H2'	117.7
O5—C5—C6	112.73 (16)	C1—O1—H1	109.5
N2—C6—C5	108.19 (15)	C5—O5—H5	109.5
			
O2—C1—C2—N1	−5.6 (3)	N2—C7—C8—Cl2	−3.8 (2)
O1—C1—C2—N1	174.91 (17)	O3—C3—N1—C2	−4.0 (3)
O3—C3—C4—Cl1	177.13 (15)	C4—C3—N1—C2	176.34 (18)
N1—C3—C4—Cl1	−3.2 (3)	C1—C2—N1—C3	−171.64 (17)
O4—C5—C6—N2	−3.7 (3)	O6—C7—N2—C6	−2.1 (3)
O5—C5—C6—N2	176.72 (16)	C8—C7—N2—C6	177.35 (17)
O6—C7—C8—Cl2	175.65 (14)	C5—C6—N2—C7	−174.44 (17)

### Hydrogen-bond geometry (Å, º)

**Table:**

*D*—H···*A*	*D*—H	H···*A*	*D*···*A*	*D*—H···*A*
N1—H1′···Cl1	0.86	2.44	2.9422 (18)	118
N1—H1′···O2	0.86	2.24	2.618 (2)	106
N2—H2′···Cl2	0.86	2.46	2.9450 (18)	116
N2—H2′···O4	0.86	2.22	2.619 (2)	108
O1—H1···O3^i^	0.82	1.84	2.647 (2)	166
O5—H5···O6^ii^	0.82	1.85	2.657 (2)	167
C4—H4*B*···O2^ii^	0.97	2.57	3.080 (3)	113
C8—H8*B*···O4^i^	0.97	2.57	3.099 (3)	114

Symmetry codes: (i) *x*, *y*+1, *z*; (ii) *x*, *y*−1, *z*.
